# Layer matching epitaxy of NiO thin films on atomically stepped sapphire (0001) substrates

**DOI:** 10.1038/srep14385

**Published:** 2015-09-24

**Authors:** Ryosuke Yamauchi, Yosuke Hamasaki, Takuto Shibuya, Akira Saito, Nobuo Tsuchimine, Koji Koyama, Akifumi Matsuda, Mamoru Yoshimoto

**Affiliations:** 1 Tokyo Institute of Technology, Yokohama 226-8502, Japan; 2 Osaka University, Osaka 565-0871, Japan; 3 Toshima Manufacturing Co., Ltd., Higashimatsuyama, Saitama 355-0036, Japan; 4 Namiki Precision Jewel Co., Ltd., Adachi, Tokyo 123-8511, Japan

## Abstract

Thin-film epitaxy is critical for investigating the original properties of materials. To obtain epitaxial films, careful consideration of the external conditions, i.e. single-crystal substrate, temperature, deposition pressure and fabrication method, is significantly important. In particular, selection of the single-crystal substrate is the first step towards fabrication of a high-quality film. Sapphire (single-crystalline α-Al_2_O_3_) is commonly used in industry as a thin-film crystal-growth substrate, and functional thin-film materials deposited on sapphire substrates have found industrial applications. However, while sapphire is a single crystal, two types of atomic planes exist in accordance with step height. Here we discuss the need to consider the lattice mismatch for each of the sapphire atomic layers. Furthermore, through cross-sectional transmission electron microscopy analysis, we demonstrate the uniepitaxial growth of cubic crystalline thin films on bistepped sapphire (0001) substrates.

Nickel oxide (NiO) with an NaCl-type structure is a wide-bandgap antiferromagnetic p-type semiconductor with excellent stability in air, good crystallinity and transparency. The multifunctional properties of NiO can be used in exchange biased systems^1^, UV detectors^2^, high-durability hole transport layers in organic solar cells^3^, electrochromic devices^4^ and UV-light-emitting diodes^5^, among other applications. The development of NiO nanostructured materials has also stimulated worldwide interest in their use as electrode materials for lithium ion batteries^6^,^7^,^8^, electrochemical supercapacitors^9^ and catalysts for carbon nanotube formation^10^ because of their high performance. Many efforts have been focused on the preparation of various NiO nanostructures, such as nanowalls^6^, nanoflake arrays^7^, nanosheets^8^, nanocrystals^9^, flower-shaped architectures^10^, nanocubes^11^ and threading dislocation-introduced NiO films^12^. Effectively controlling the size, morphology and structure of NiO nanomaterials for applications involving morphology-dependent properties is imperative.

Sapphire is used in industry as a thin-film crystal-growth substrate because of the mass-productivity as large-diameter wafers, thermal and chemical stability and transparency. Furthermore, atomically flat surfaces referred to as step-and-terrace structures are formed on mirror-polished sapphire (0001) substrates upon annealing in air at approximately 1273 K^13^. The structures comprise atomically smooth terraces separated by straight atomic steps with a 1/6^th^ height of the c-axis in the sapphire unit cell. The substrate terrace width *w* is defined as *w* *=* *h/*tan *θ*, where *h* and *θ* are the step height and miscut angle of the sapphire, respectively. If *w* >1 [nm], the off-angle *θ* is inversely proportional to *w* for a single step of the sapphire (0001) plane, and the step direction is determined by the off-direction to which the straight steps are formed perpendicularly. No deterioration of the step-and-terrace structure of sapphire (0001) substrates occurs at room temperature in air. The formation of the step-and-terrace structure suggests that atoms on the sapphire surface migrate to minimise the surface energy at less than a half of the sapphire melting point (2326 K).

Substrate surface structures can affect crystallinity and morphology of thin-films^14^,^15^,^16^. Previously, we observed the formation of periodic nanogroove arrays on room-temperature pulsed-laser-deposited NiO (111) epitaxial thin films grown on atomically stepped sapphire (0001) substrates via post-thermal annealing in air, but the mechanism remained under discussion^17^. The structures were definitely related to the atomic steps of the sapphire (0001) substrates because the nanogroove periodicity corresponded well with the sapphire (0001) substrate terrace width^17^. The post-thermal annealing may have caused reconstruction to the most stable structure, i.e. periodic nanogroove arrays. It is needed to reveal the driving force for formation of the periodic nanogroove array without the coalescence of adjacent film terraces.

Here, the mechanism for NiO periodic straight nanogroove array formation is explained using a new concept ‘layer matching epitaxy (LME) model’ in which the lattice mismatch between the thin film and the substrate is postulated by the in-plane atomic geometry for each topmost surface layer. The LME model was substantiated by transmission electron microscopy (TEM) results and led to the fabrication of bistepped sapphire (0001) substrates.

Figure 1(a) presents an atomic force microscopy (AFM) surface image of an NiO thin film on a 0.22-nm-high stepped sapphire (0001) substrate after rapid thermal annealing (RTA) at 1373 K for 60 s. A periodic straight nanogroove array was formed over the RTA-treated NiO thin-film surface. The cross-sectional scanning transmission electron microscopy bright field (STEM-BF) image shown in Fig. 1(b) revealed that V-shaped and terraced-rice-field-shaped grooves alternately existed on the NiO thin-film surface. In the magnified STEM images presented in Fig. 1(c,d), periodic linear defects stretching over the deepest part of a nanogroove towards the substrate interface can be more clearly observed. The STEM-energy dispersive X-ray spectrometry (EDX) results shown in Fig. 1(e) further revealed that atomic diffusion at the interface was virtually negligible, even though the sample was annealed at 1373 K for 60 s. Furthermore, the cross-sectional transmission electron microscopy and selected-area electron diffraction (TEM-SAED) results shown in Fig. 1(f) indicated that the NiO diffraction patterns were mirror-symmetrical between the next NiO crystal domains, with the linear defect as the mirror plane. Finally, the high-resolution transmission electron microscopy (HRTEM) images presented in Fig. 1(g,h) revealed that the sapphire substrate under the deepest part of the nanogroove was dented, and after RTA, the substrate did not exhibit the 0.22-nm-high atomic steps observed at the interface between the NiO (111) epitaxial thin film and sapphire (0001) substrate prior to RTA. Considering the SAED patterns shown in Fig. 1(f), it can be concluded that the V-shaped groove comprised NiO (002) facets and the terraced-rice-field-shaped grooves comprised (111) and (11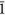) facets. Therefore, the linear defects were planar defects that occurred at the NiO twin domain boundaries near the atomic steps, and these NiO twin boundaries were the driving force for the formation of the periodic nanogroove array without coalescence of the adjacent film terraces.

These results suggest that the NiO crystal grains were uniepitaxially grown on each terrace of the 0.22-nm-high stepped sapphire (0001) substrate. In fact, after RTA at 973 K for 60 s, two types of 180°-rotated triangular crystalline domains were alternately grown on each film terrace divided by the nanogrooves, and the two types of triangular crystals were not mixed on one film terrace^17^. It is well known that NiO thin films grow biepitaxially on sapphire (0001) substrates with the crystallographic orientation relationships NiO(111)[1
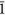
0]||sapphire(0001)[01
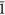
0] and NiO(111)[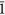
10]||sapphire(0001)[01
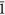
0], even for deposition at room temperature^18^,^19^. Consequently, NiO thin-film epitaxy on sapphire has been conventionally explained using the domain matching epitaxy (DME) theory in which matching between the film and substrate is estimated using the integral multiple of the lattice planes^18^,^19^,^20^,^21^. For instance, the lattice mismatch between NiO (ICDD card No. 47–1049) and sapphire (ICDD card No. 46–1212) can be calculated as follows:


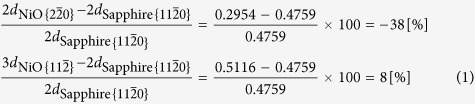


where *d* denotes lattice plane determined by three or four Miller indices (hkl) or (hkil). However, the mirror-symmetrical SAED patterns observed in Fig. 1(f) cannot be interpreted using the DME concept, because there is no difference in the mismatches before and after rotating the NiO (111) plane by 180°. There must be a reason why the two different types of 180°-rotated triangular crystalline domains were alternately grown on each film terrace divided by the nanogrooves. Therefore, the mismatch between the NiO film and the sapphire substrate was considered using a new concept based on a layer matching epitaxy (LME) model in which the misfit between the thin film and the substrate is estimated on the basis of the difference in the in-plane atomic geometry for each topmost surface layer. In the stepped sapphire (0001) plane, the oxygen layer comprises equilateral triangles, each with a side length of 0.2525 nm, surrounded by six equilateral triangles, each with 0.2867-nm-long sides that are tilted by 3.87°, as shown in Fig. 2(a). Furthermore, the oxygen sublattices are mirror-symmetrical between the adjacent terraces, with a 1/6^th^ height of the c-axis in the sapphire unit cell. On the other hand, the NiO (111) atomic layer comprises regular rhombuses with 0.2954-nm-long sides in which the Ni layer and the O layer are alternately stacked [Fig. 2(b)]. Therefore, NiO is epitaxially grown on the 0.22-nm-high stepped sapphire (0001) plane to match the mirror-symmetrical oxygen layer between the adjacent substrate terraces. In short, although sapphire is a single crystal, two types of terraces exist in accordance with the step height. For example, six-fold biepitaxy is observed rather than three-fold epitaxy for NiO deposited on a 0.22-nm-high stepped sapphire (0001) plane due to the presence of the two types of mirror-symmetrical oxygen sublattice in the sapphire (0001) plane. Therefore, the lattice mismatch for all of the sapphire atomic layers should be considered.

Formation of grooved nanostructures was then achieved on the surfaces of Li_x_Ni_1−x_O thin films (x < 0.5) deposited on atomically stepped sapphire (0001) substrates after RTA at 1373 K for 60 s. The depth of the nanogroove array was five times greater than that on the annealed nondoped NiO film [Fig. 3(a)]. This deeper nanogroove formation may be due to the low boiling point of Li. The planar defects near the atomic steps of the sapphire (0001) substrate diffused because of thermal annealing with simultaneous Li migration in all directions, resulting in restructuring and deeper nanogroove formation. The magnified TEM image shown in Fig. 3(b) revealed that the lattice spacing near the thin-film surface was 0.24 nm and corresponded to the interplanar spacing of NiO (111), while that near the interface was 0.47 nm. In the STEM-EDX images presented in Fig. 3(c), an interface reaction layer different from that observed in the annealed pure NiO thin film [compare Fig. 3(c) with Fig. 1(e)] can be seen. Then, the STEM-electron energy loss spectroscopy (EELS) spectra [Fig. 3(d)] indicated that this interface reaction layer comprised Al, Ni, O and diffused Li. Thus, while sapphire has high thermal durability, it was affected by diffused Li at 1373 K in just 60 s. It should also be noted that (i) the dark areas in the NiO thin film in the STEM-HAADF image shown in Fig. 3(c) represent the lower-density areas and (ii) the Li completely disappeared from the NiO thin film. Therefore, mirror-symmetrical NiO crystal domains were formed in the RTA-treated LiNiO thin film, and the Li reacted with the sapphire (0001) substrate to generate a lithium nickel aluminate phase. Wide area surface observation via scanning electron microscopy (SEM) revealed a uniform array of straight nanogrooves over a large area [Fig. 3(e)], leading to a rainbow-coloured interference pattern on the surface of the film [Fig. 3(f)]. Although the reflectivity was less than 20% [Fig. 3(g,h)]^22^, the reflective angular profiles of these rainbow-coloured films corresponded well with the grating equation sin *α*−sin *β* = *λ*/*d*, where *α*, *β*, *λ* and *d* are incident and diffraction angles, wavelength of incident light and groove pitch, respectively. Furthermore, manifestation of structural colour suggested that the NiO thin film was grown in accordance with the LME model uniformly over the entire substrate, despite heavy Li doping.

According to the mechanism for nanogroove formation based on the LME model, NiO thin films should be uniepitaxially grown on bistepped sapphire (0001) substrates. However, to the best of our knowledge, the fabrication of sapphire (0001) substrates that are bistepped over the entire surface has not been previously reported. Thus, step formation was examined by systematically varying the annealing temperature at a heating rate of 10 K/min (Fig. 4). It was found that step formation in the sapphire (0001) substrate occurred at 973 K, and the step linearity was improved at 1273 K. After annealing at temperatures above 1473 K for 3 h, the atomic steps of the sapphire (0001) planes bunched up with random heights, and the bunched step array was not straight. Furthermore, the step heights were not always equal to an integral multiple of the sapphire (0006) planes, i.e. 0.55-nm-high steps were found. Therefore, the sapphire (0001) surface after annealing for 3 h at temperatures between 1473 K and 1573 K was mixed with the Al and O layers. Furthermore, the step array of the sapphire (0001) surface was nearly straight after annealing at 1673 K for 3 h, but the step heights were not uniform in the measurement area [compare Fig. 4(j) with (k)], and the terrace widths were irregular over 5 × 5 μm^2^ area. Notably, a bistepped sapphire (0001) surface was only partially obtained when the annealing was performed at 1473 K for 3 h with the heating rate decreased from an initial rate of 10 K/min to 1 K/min at 1373 K [Fig. 5(a)]. The bistep ratio *r*_*b*_ increased as the annealing time increased [Fig. 5(b)] and was estimated using the following equation:


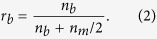


In equation (2), *n*_*b*_ and *n*_*m*_ represent the number of bisteps and monosteps, respectively. The step morphology and the cross-sectional height profile are shown in Fig. 5(c). It should be noted that bistep formation occurred only for off-angles greater than 0.13°. After deposition of NiO, the thin-film surface definitely reflected the step-and-terrace structure [Fig. 5(d)]. It is also noteworthy that a nanogroove array did not form on the surface of the NiO thin film grown on the 0.43-nm-high stepped sapphire (0001) substrate after RTA at 1373 K for 60 s, and the triangular NiO crystal grains all possessed the same orientation [Fig. 5(e)], which supported the LME model. Furthermore, an XRD φ scan of the as-deposited NiO thin film grown on the 0.43-nm-high stepped sapphire (0001) substrate revealed that the NiO thin film was grown uniepitaxially on the bistepped sapphire (0001) substrate with the crystallographic orientation relationship NiO(111)[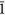
10]||sapphire(0001)[01
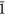
0], despite being deposited at room temperature [Fig. 5(f)].

In summary, we have discovered that NiO thin films are uniepitaxially grown on bistepped sapphire (0001) substrates in accordance with a new concept LME model which is modelled from the results that the driving force for the formation of the periodic nanogroove array without coalescence of the adjacent film is NiO twin boundaries resulting from the crystal growth behaviour that NiO is uniepitaxially grown on one terrace of 0.22-nm-high stepped sapphire (0001) plane to match the mirror-symmetrical oxygen sublattice layer between the adjacent substrate terraces, despite heavy Li doping. In future, various cubic functional thin-film materials will be grown on the bistepped sapphire (0001) substrate, and the twin boundary will decrease leading to fabrication of high-quality cubic thin films on sapphire (0001) substrate.

## Methods

NiO epitaxial thin films were grown on atomically stepped sapphire (0001) substrates at room temperature in 1.3 mPa O_2_ via pulsed laser deposition using a sintered ceramic target and a pulsed KrF excimer laser (wavelength: 248 nm, pulse duration: 20 ns, frequency: 5 Hz). The target was rotated for uniform ablation and to preclude droplet formation. During the deposition of each film, the distance between the target and the substrate was 5 cm. The film growth rate was between 0.02 and 0.03 nm/s. The surface morphologies of the films were observed using atomic force microscopy. The crystal structure and epitaxial growth of the films were examined via X-ray diffraction analysis. After deposition, the obtained NiO (111) epitaxial thin films were annealed via rapid thermal annealing (RTA) at a heating rate of 20 K/s at 1373 K for 60 s and subsequently quenched to room temperature. During RTA, the samples were annealed using a near-infrared ray collimated using a parabolic reflector to uniformly heat the samples. The microscale morphology of the RTA-treated LiNiO thin film was confirmed by scanning electron microscopy (SEM). The interface between the thin film and sapphire (0001) substrate was observed via scanning transmission electron microscopy combined with energy dispersive X-ray spectroscopy (STEM-EDX) and electron energy loss spectroscopy (STEM-EELS). The local crystal domains of the films were examined via transmission electron microscopy and selected area electron diffraction (TEM-SAED) analysis.

## Additional Information

**How to cite this article**: Yamauchi, R. *et al.* Layer matching epitaxy of NiO thin films on atomically stepped sapphire (0001) substrates. *Sci. Rep.* **5**, 14385; doi: 10.1038/srep14385 (2015).

## Figures and Tables

**Figure 1:**
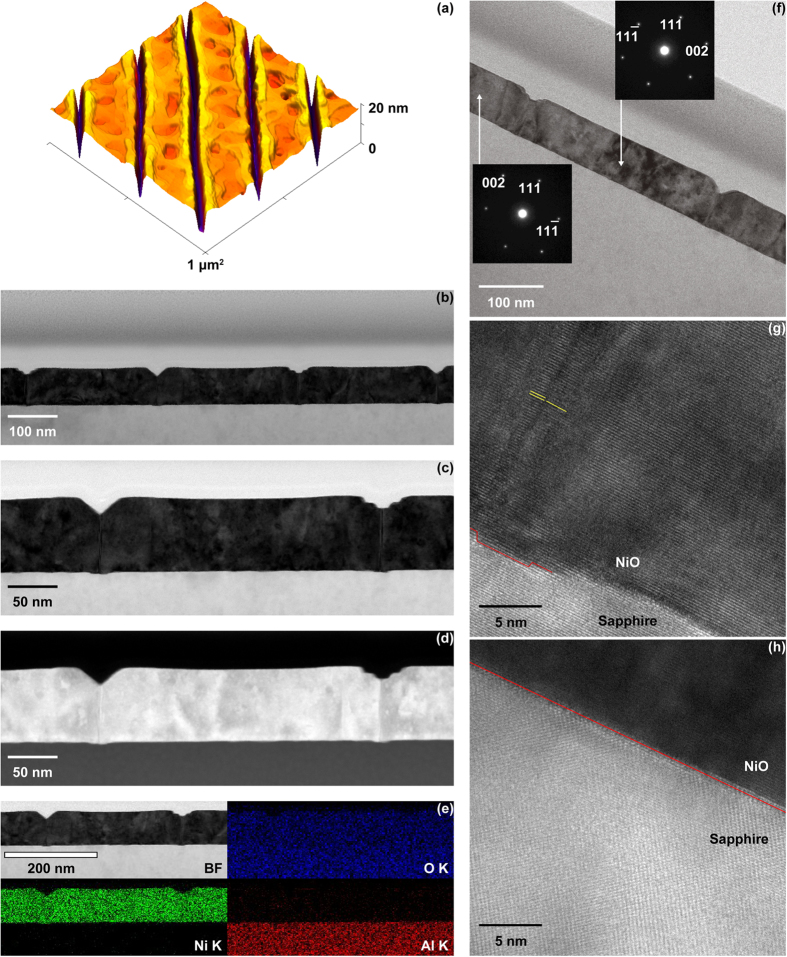
(**a**) AFM , (**b**,**c**) STEM-BF, (**d**) STEM-HAADF, (**e**) STEM-EDX and (**f**) TEM-SAED images of an NiO thin film on a 0.22-nm-high stepped sapphire (0001) substrate after RTA at 1373 K for 60 s. HRTEM images show interfaces between the NiO and sapphire (**g**) under the deepest part of the nanogroove after RTA at 1373 K for 60 s and (**h**) near the 0.22-nm-high atomic step before RTA.

**Figure 2:**
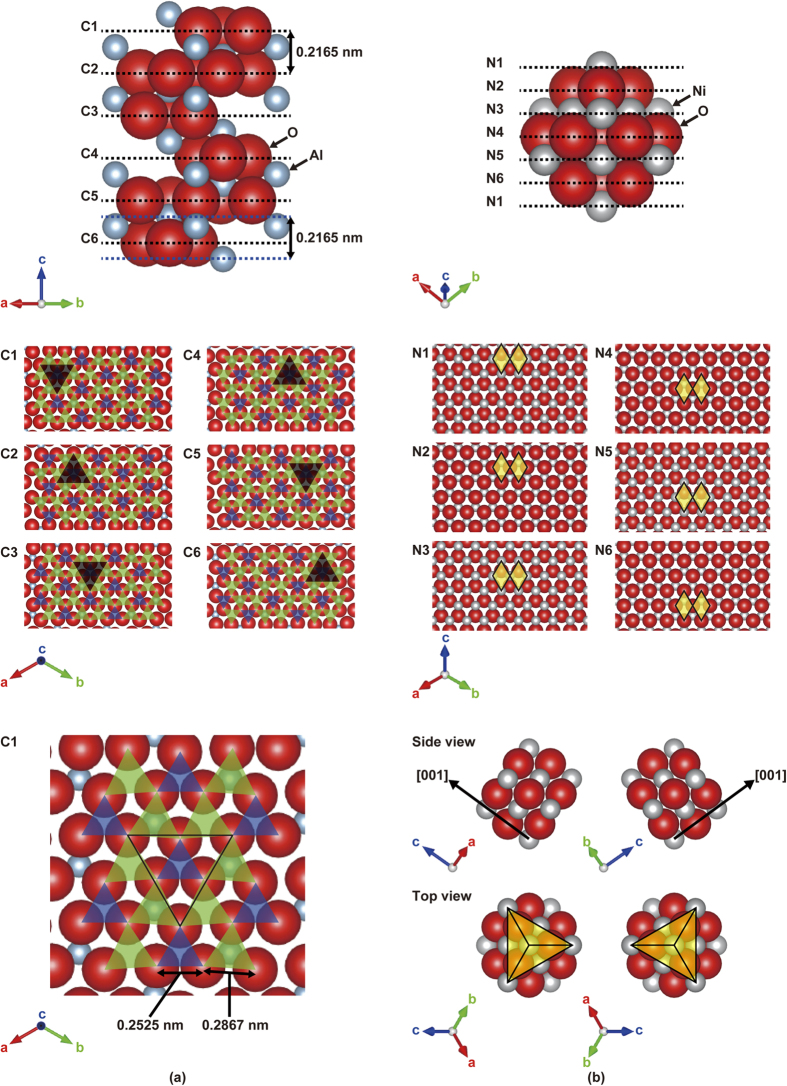
Hard-sphere surface model of (a) sapphire and (b) NiO. The green and slightly smaller blue triangles in the sapphire denote the equilateral triangular oxygen sublattices of sapphire with side lengths of 0.2867 nm and 0.2525 nm, respectively. The yellow regular rhombuses in the NiO represent NiO (111) structural units with a side length of 0.2954 nm. The triangular line in the sapphire indicates that the green triangles are tilted at 3.87°and share a corner with the blue triangles. The side and top views of NiO reveal that the difference between the two types of NiO domains is related to the atom stacking direction, i.e. the NiO[001] direction, which is in agreement with the direction that the yellow rhombuses move.

**Figure 3:**
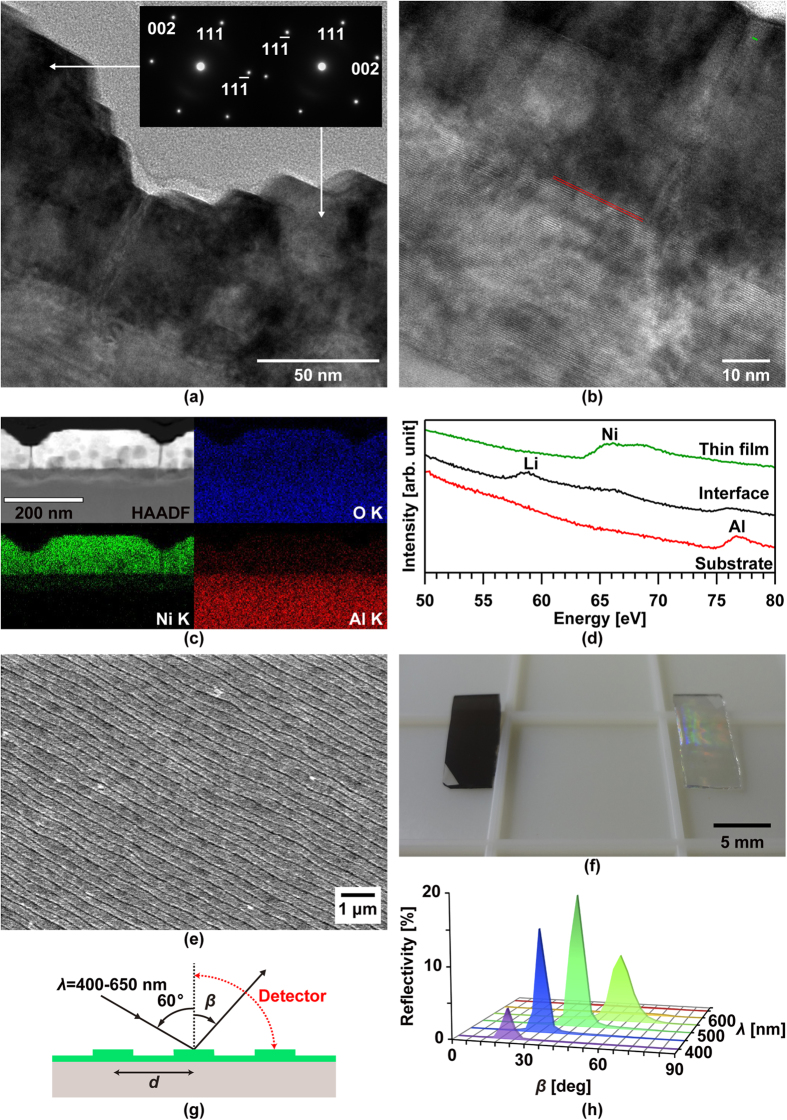
(**a**) TEM-SAED image, (**b**) TEM image, (**c**) STEM-EDX images, (**d**) STEM-EELS spectra and (**e**) top-view SEM image of an Li_0.5_Ni_0.5_O thin film on a 0.22-nm-high stepped sapphire (0001) substrate after RTA at 1373 K for 60 s. The TEM image (**b**) shows the magnified area in (**a**) containing the interface reaction layers. The green and red lines in (**b**) indicate lattice planes with spacings of 0.24 nm and 0.47 nm. The photograph in (**f**) shows (left) the as-deposited and (right) RTA-treated Li_0.5_Ni_0.5_O thin film on a 0.22-nm-high stepped sapphire (0001) substrate. The optical diffraction properties of the sample (f, right side) were obtained at the position shown in (**g**) and presented in (**h**).

**Figure 4:**
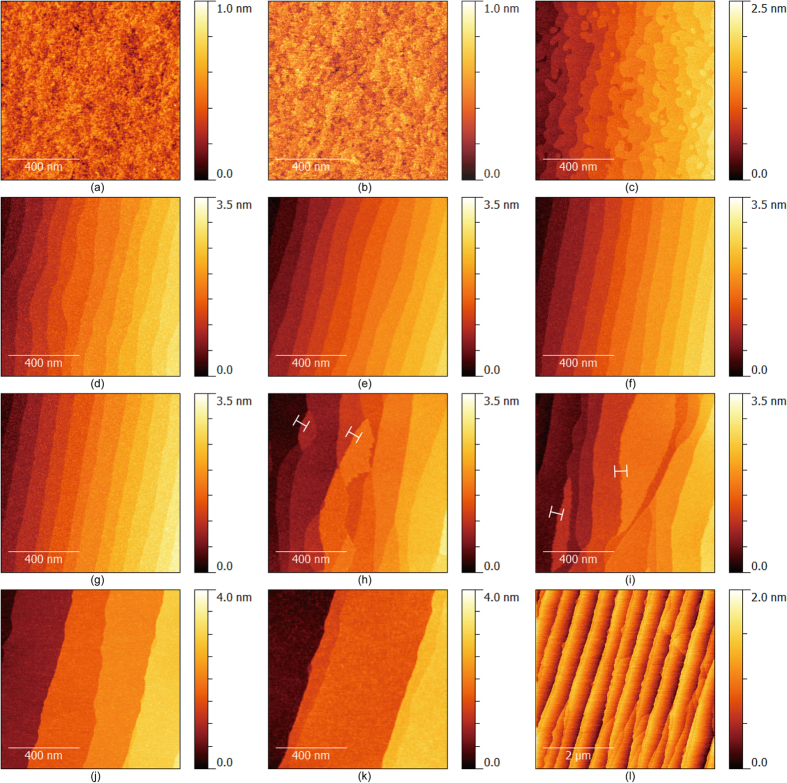
Sapphire (0001) substrate surface images. Mirror-polished substrates (**a**) untreated and annealed for 3 h at (**b**) 873 K, (**c**) 973 K, (**d**) 1073 K, (**e**) 1173 K, (**f**) 1273 K, (**g**) 1373 K, (**h**) 1473 K, (**i**) 1573 K and (**j–l**) 1673 K. The images were taken over an area of (**a–k**) 1 × 1 μm^2^ and (l) 5 × 5 μm^2^. All of the cross-sectional heights at the lines in (**h**,**i**) were 0.55 nm.

**Figure 5:**
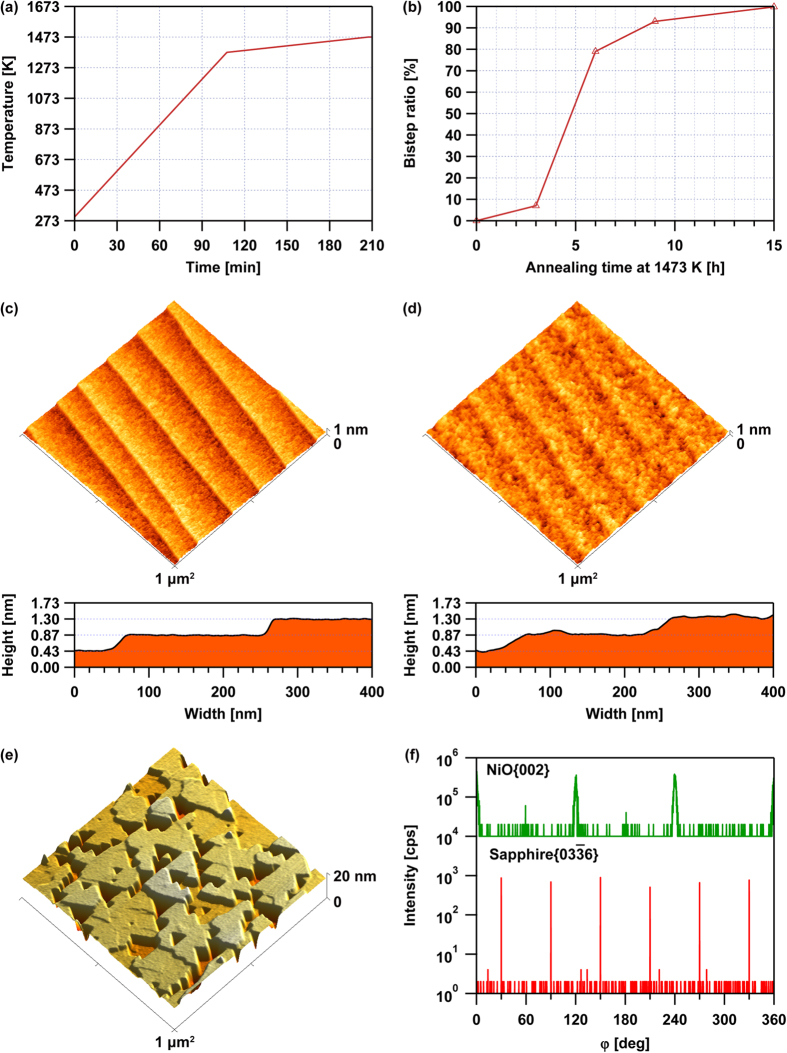
Bistepped sapphire (0001) substrates were fabricated in a conventional annealing furnace using the annealing rate shown in (**a**). The relationship between the annealing time and the sapphire bistep ratio for a sapphire off-angle of 0.13° is displayed in (**b**). A surface image and cross-sectional height profile of the bistepped sapphire (0001) substrate are presented in (**c**), and the step heights corresponded well to 1/3^rd^ the height (0.433 nm) of the c-axis in the sapphire unit cell. An NiO thin film grown on the substrate shown in (**c**) reflected the step-and-terrace structure, as can be seen in (**d**), suggesting the layer-by-layer growth of NiO on the sapphire (0001) plane. After RTA at 1373 K for 60 s, the step-and-terrace structure seen in (**d**) changed to that shown in (**e**). The XRD φ-scan results that can be seen in (**f**) were obtained by tilting the specimen shown in (**d**) in order to detect the NiO{002} and sapphire {03
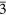
6} peaks.

## References

[b1] Nogués J. & Schuller I. K. Exchange bias. J. Magn. Magn. Mater. 192, 203–232 (1999).

[b2] Ohta H. *et al.* Fabrication and photoresponse of a pn-heterojunction diode composed of transparent oxide semiconductors, p-NiO and n-ZnO. Appl. Phys. Lett. 83, 1029–1031 (2003).

[b3] Jung J. *et al.* Stability enhancement of organic solar cells with solution-processed nickel oxide thin films as hole transport layers. Sol. Energ. Mat. Sol. Cells 102, 103–108 (2012).

[b4] Ren Y. *et al.* The coloration and degradation mechanisms of electrochromic nickel oxide. Sol. Energ. Mat. Sol. Cells 116, 83–88 (2013).

[b5] Deng R. *et al.* Ultraviolet electroluminescence from n-ZnO/p-NiO heterojunction light-emitting diode. J. Lumin. 134, 240–243 (2013).

[b6] Varghese B. *et al.* Fabrication of NiO nanowall electrodes for high performance lithium ion battery. Chem. Mater. 20, 3360–3367 (2008).

[b7] Wu H. *et al.* Aligned NiO nanoflake arrays grown on copper as high capacity lithium-ion battery anodes. J. Mater. Chem. 22, 9821–19825 (2012).

[b8] Huang Y. *et al.* Self-assembly of ultrathin porous NiO nanosheets/graphene hierarchical structure for high-capacity and high-rate lithium storage. J. Mater. Chem. 22, 2844–2847 (2012).

[b9] Zhang X. J. *et al.* Synthesis of porous NiO nanocrystals with controllable surface area and their application as supercapacitor electrodes. Nano Res. 3, 643–652 (2010).

[b10] Zhou Q. L., Gu F. & Li C. Self-organized NiO architectures: Synthesis and catalytic properties for growth of carbon nanotubes. J. Alloy. Compd. 474, 358–363 (2009).

[b11] Chen D. P. *et al.* Growth mechanism and magnetic properties of highly crystalline NiO nanocubes and nanorods fabricated by evaporation. Cryst. Growth Des. 12, 2842–2849 (2012).

[b12] Sugiyama I. *et al.* Ferromagnetic dislocations in antiferromagnetic NiO. Nat. Nanotechnol. 8, 266–270 (2013). 23524441 10.1038/nnano.2013.45

[b13] Yoshimoto M. *et al.* Atomic-scale formation of ultrasmooth surfaces on sapphire substrates for high-quality thin-film fabrication. Appl. Phys. Lett. 67, 2615–2617 (1995).

[b14] Itaka K., Myojin N., Yamashiro M., Yamaguchi J. & Koinuma H. Molecular beam epitaxy of highly oriented pentacene thin films on an atomically flat sapphire substrate. Jpn. J. Appl. Phys. 44, 6249–6251 (2005).

[b15] Wang Y. *et al.* Effects of sapphire substrate annealing on ZnO epitaxial films grown by MOCVD. Appl. Surf. Sci. 253, 1745–1747 (2006).

[b16] Brockman J., Samant M. G., Roche K. P. & Parkin S. S. P. Substrate-induced disorder in V_2_O_3_ thin films grown on annealed c-plane sapphire substrates. Appl. Phys. Lett. 101, 051606 (2012).

[b17] Yamauchi R. *et al.* Influence of rapid thermal annealing and substrate terrace width on self-organizing formation of periodic straight nanogroove array on NiO(111) epitaxial thin film. Jpn. J. Appl. Phys. 51, 06FF16 (2012).

[b18] Lee J. H., Kwon Y. H., Kong B. H., Lee J. Y. & Cho H. K. Biepitaxial growth of high-quality semiconducting NiO thin films on (0001) Al_2_O_3_ substrates: Microstructural characterization and electrical properties. Cryst. Growth Des. 12, 2495–2500 (2012).

[b19] Anli Y. *et al.* Lattice spacings and domain sizes of room-temperature epitaxial Li_x_Ni_1−x_O (0 ≤ x ≤ 0.48) thin films grown on ultra-smooth sapphire substrates. Appl. Surf. Sci. 320, 787–790 (2014).

[b20] Dutta T., Gupta P., Gupta A. & Narayan J. Effect of Li doping in NiO thin films on its transparent and conducting properties and its application in heteroepitaxial p-n junctions. J. Appl. Phys. 108, 083715 (2010).

[b21] Narayan J. Recent progress in thin film epitaxy across the misfit scale. Acta Mater. 61, 2703–2724 (2013).

[b22] Yokomori K. Dielectric surface-relief gratings with high diffraction efficiency. Appl. Optics 23, 2303–2310 (1984). 10.1364/ao.23.002303 18212995

